# Hemoadsorption of cytokines by CytoSorb filter: a simulation study without human factor—pilot is the difference

**DOI:** 10.1186/s13054-019-2716-y

**Published:** 2020-01-13

**Authors:** Rashid Nadeem, Zainab Alameeer Obaida, Sahish Kamat

**Affiliations:** Dubai Hospital, P O Box 7272, Dubai, United Arab Emirates

To the Editor:

We read with great interest the article published in a recent issue of *Critical Care* by Brouwer et al. [1]. Authors showed the beneficial effect of addition of CytoSorb filter to continuous renal replacement therapy (CRRT) for hemoadsorption of cytokines in management of septic shock. We would like to raise few points which may be important for authors and readers of the article.

This was an investigator-initiated retrospective study of non-consecutive patients, treated by Cytosorb plus CRRT versus CRRT alone. This study was non-randomized, and analysis was not “intention to treat”.

We believe both group populations were significantly different (disproportionately higher number of patients in control group with diabetes, hypertension, heart failure, and renal disease and nonsurgical patients) and difference in mortality may just be the result from this difference rather than intervention for the following reasons. Authors describe inclusion of all factors in regression model for determination for its association with treatment allocation; they should acknowledge that investigator initiation itself is a significant factor for bias which cannot be adjusted. Investigator-led management group will likely to have better attention and management. Relatively imperfect attention of physician taking care of patients in control group is reflected by the inappropriate assignment of renal failure as sepsis in 60 out of 109 patients.

Authors used propensity score, though treatment effect made using propensity-score matching are valid only if a matched sample of treated and untreated subjects has similar distributions of measured baseline covariates [2]. Figure 2 suggests the difference in mortality only appears after application of “stabilized inverse probability of treatment weights (sIPTW)” methods to correct the imbalance between the groups.

Additionally, there were factors not provided: difference in fluid management, time to start antibiotics, and ventilator strategy. These factors influence the course of disease and mortality.

Lack of information about ultrafiltration rates also made it difficult to compare the quality of CRRT between groups.

Without randomization and matching, investigator can erroneously assign patients with high unadjusted SOFA scores on admission but greatest potential for improvement (younger surgical patients without chronic illness) to the treatment group. Hence, the observed mortality is lower than expected. They used sIPTW to correct the imbalance between the groups to negligible levels for example for lactate (85% reduced to 10%), and noradrenaline (110% to 10%). Underestimation of the variance may produce inappropriately narrow confidence intervals and leads to false rejection of the null hypothesis because of inflated sample sizes [3].

This analysis may be more effective in removing the difference between variables between the cases and the controls than CytoSorb which is effective in removing the cytokines.

Lastly, simulation testing ignores the most important factor “human factor,” the same factor which saved 155 lives of the US airways passengers when pilot Sully landed his plane on river Hudson after both engines failed in 2009. Simulation showed that he should have turned back to LaGuardia airport where he could have safely landed. Facts review determined that if he had acted like simulation, everyone would have died.

To summarize, this study has multiple issues as outlined above, though it is a good start. No adverse effects reported so we can expect a well-designed prospective, randomized control trial to evaluate the impact of Cytosorb on clinical outcomes like mortality.

## Authors’ response

Willem P. Brouwer, Servet Duran, Martijn Kuijper, Can Ince

To the Editor:

We thank Nadeem et al. for their interest in our work and comments regarding our publication and the opportunity to further clarify the complex analysis in our study.

They raise several questions with regard to the comparability of the patients in our study, as well as not including a human factor. Although we appreciate the interesting comparison of the “Hudson River plane crash,” we respectfully believe this analogy has no relevance to our study. We agree however that because of the retrospective nature of our study, a human factor cannot be completely eradicated. Yet, by applying the “inverse probability of treatment weight (IPTW)” method, the patient characteristics at the start of therapy are comparable as shown in Table 1 and by the supplementary figure [1, 2, 4, 5].

IPTW is a methodology accepted in many key note publications. It can be applied to estimate causal treatment effects in situations where the treatment was non-randomized and confounding factors may be present. The propensity score (i.e., the probability of being treated, given the set of confounders) is constructed and used as a weighting factor in order to create a comparable set of patients. This means that, after applying the weights, the distribution of the patient characteristics at the start of therapy is now *independent of the treatment assignment*, such as is the case in a randomized controlled trial [1, 2, 4, 5].

In contrast to the statements in their letter, after applying IPTW, our patients had comparable distributions of covariates at the start of therapy. Moreover, Nadeem et al. wrongly claim that an unweighted regression analysis was performed to produce the final results, and also falsely claim that the sample size was inflated by using this method. The weights were normalized using the estimated marginal means of the propensity score, and as a result, there is not an inflated sample size and the confidence intervals are not falsely narrowed. Data with respect to the IPTW and the distribution of the covariates can be found in the supplementary figure [1].

The observed versus expected mortality rates were compared according to the SOFA score within, but not between groups. Again it should be stressed that a comparison of two completely different patient groups was not performed. Indeed, we agree that a *p* value in Figure 2B (no sIPTW) can lead to some confusion, but it was printed to show the difference in mortality between the groups in case they were not weighted (i.e., unadjusted for confounders). Lastly, Nadeem et al. summarize a list of limitations of our study. We would kindly like to refer the authors to the discussion section of our paper where these limitations had already been thoroughly considered [1].

## Data Availability

Not applicable.
